# A randomized controlled trial of postoperative rehabilitation using digital healthcare system after rotator cuff repair

**DOI:** 10.1038/s41746-023-00842-7

**Published:** 2023-05-23

**Authors:** Ga Yang Shim, Eun Hye Kim, Yun Jeong Baek, Won Kee Chang, Bo Ram Kim, Joo Han Oh, Jong In Lee, Ji Hye Hwang, Jae-Young Lim

**Affiliations:** 1 Department of Physical and Rehabilitation Medicine, Kyung Hee University College of Medicine, Kyung Hee University Hospital, Seoul, Republic of Korea; 2 Department of Rehabilitation Medicine, Seoul National University Bundang Hospital, Seongnam, Gyeonggi Republic of Korea; 3 Department of Orthopaedic surgery, Seoul National University Bundang Hospital, Seongnam, Gyeonggi Republic of Korea; 4 Department of Rehabilitation Medicine, Seoul St. Mary’s Hospital, College of Medicine, The Catholic University of Korea, Seoul, Republic of Korea; 5 Department of Physical and Rehabilitation Medicine, Sungkyunkwan University School of Medicine, Seoul, Republic of Korea; 6 Department of Rehabilitation Medicine, Seoul National University College of Medicine, Seoul, Republic of Korea

**Keywords:** Rehabilitation, Orthopaedics, Geriatrics, Rehabilitation

## Abstract

A digital healthcare system based on augmented reality (AR) has promising uses for postoperative rehabilitation. We compare effectiveness of AR-based and conventional rehabilitation in patients after rotator cuff repair (RCR). This study randomly allocates 115 participants who underwent RCR to digital healthcare rehabilitation group (DR group) and conventional rehabilitation group (CR group). The DR group performs AR-based home exercises using UINCARE Home+, whereas the CR group performs brochure-based home exercises. The primary outcome is a change in the Simple Shoulder Test (SST) score between baseline and 12 weeks postoperatively. The secondary outcomes are the Disabilities of the Arm, Shoulder and Hand (DASH) score; Shoulder Pain And Disability Index (SPADI) score; EuroQoL 5-Dimension 5-Level (EQ5D5L) questionnaire score; pain; range of motion (ROM); muscle strength; and handgrip strength. The outcomes are measured at baseline, and at 6, 12, and 24 weeks postoperatively. The change in SST score between baseline and 12 weeks postoperatively is significantly greater in the DR group than in the CR group (*p* = 0.025). The SPADI, DASH, and EQ5D5L scores demonstrate group×time interactions (*p* = 0.001, = 0.04, and = 0.016, respectively). However, no significant differences over time are observed between the groups in terms of pain, ROM, muscle strength, and handgrip strength. The outcomes show significant improvement in both groups (all *p* < 0.001). No adverse events are reported during the interventions. AR-based rehabilitation shows better improvement in terms of shoulder function after RCR compared to conventional rehabilitation. Therefore, as an alternative to the conventional rehabilitation, the digital healthcare system is effective for postoperative rehabilitation.

## Introduction

Rotator cuff tear is one of the most common shoulder disorders. It affects almost 30% of individuals older than 60 years, its prevalence is nearly doubles to 60% in individuals aged 80 years^1^. The predominant symptoms are pain and functional limitations in activities of daily living^2^. The first-line treatment for a rotator cuff tear is conservative management^3^; surgical rotator cuff repair (RCR) is required in cases of failed conservative management^4^.

Rehabilitation is essential for a good functional outcome after RCR^5^. Postoperative rehabilitation is initiated with immobilization and passive movements for 4–6 weeks. Active movements and resumption of light work are recommended at 7–12 weeks. Progressive functional recovery, including the ability to perform physical activities and sports, is achieved from 13 weeks onward^6^. Several attempts have been made to perform postoperative rehabilitation at patients’ homes; the need for home-based postoperative rehabilitation has particularly increased over the past few years^7,8^. During the COVID-19 pandemic, telerehabilitation was urgently required to minimize the infection risk and physical contact. There was no significant difference in functional outcomes between telerehabilitation and face-to-face rehabilitation, and telerehabilitation was more cost-effective^9,10^. However, there are technical limitations to the use of telerehabilitation, particularly in terms of its implementation, the need for a stable internet connection, and ease of use^11^.

With advances in augmented reality (AR), it has been adopted for use in clinical medicine. AR combines artificial and real reality by creating images based on digital information and implementing them in real environments. AR-based rehabilitation allows real-time interaction, increasing participation by patients, and improves physical outcomes^12^. For these reasons, AR-based rehabilitation has been evaluated for various uses, mainly including neurorehabilitation^13,14^. However, few studies have evaluated AR-based postoperative rehabilitation of the upper limb, particularly for patients with RCR.

The aim of this study was to compare the effectiveness of rehabilitation using an AR-based digital healthcare system and conventional rehabilitation for patients after RCR.

## Results

**Fig. 1:**
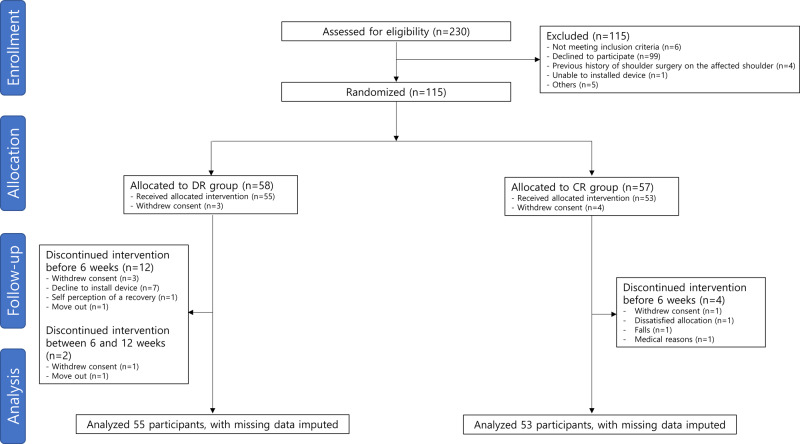
Consolidated standards of reporting trials flow diagram. DR group digital healthcare rehabilitation group; CR group conventional rehabilitation group.

A total of 230 patients who underwent RCR were screened for eligibility. Of those, half were excluded; most of the excluded patients declined to participate, 10 did not fulfil the inclusion or exclusion criteria, 1 was unable to install device, and 5 provided other reasons. Finally, 115 participants were randomized into the DR (*n* = 58) and CR (*n* = 57) groups. The intention-to-treat analysis included 108 participants, after excluding the 7 who withdrew consent before the intervention was initiated. During intervention, 14 and 4 participants in the DR and CR groups dropped out of the study. The Consolidated Standards of Reporting Trial flow diagram is presented in Fig. 1.

### Baseline characteristics

There were no significant differences in the baseline demographics and clinical variables between the DR and CR groups (Table 1). The right shoulder was more commonly affected in both groups. No adverse events were experienced by participants in either group during the interventions.

**Table 1:** Baseline characteristics of participants.

	DR group (*n* = 58)	CR group (*n* = 57)	*p* value^a^
*Demographics*			
Age (years)	63.9 ± 6.2	63.7 ± 6.7	0.871
Sex			0.618
Male	24 (41.4%)	21 (36.8%)	
Female	34 (58.6%)	36 (63.2%)	
Weight (kg)	64.7 ± 11.1	64.6 ± 10.5	0.956
Height (cm)	162.2 ± 7.3	160.0 ± 7.6	0.126
Body mass index (kg/m^2^)	24.5 ± 3.3	25.2 ± 3.4	0.296
Involved site			0.734
Right	39 (67.2%)	40 (70.2%)	
Left	19 (32.8%)	17 (29.8%)	
*Clinical information*			
Tear category^b^			0.273
Small	14 (24.1%)	7 (12.3%)	
Medium	27 (46.6%)	33 (57.9%)	
Large to massive	17 (29.3%)	17 (29.8%)	
Tear size (cm)			
Retraction	2.1 ± 1.0	2.4 ± 0.9	0.153
Anteroposterior dimension	1.7 ± 0.8	1.8 ± 0.9	0.803
Thickness of the acromion (mm)	8.36 ± 1.51	8.47 ± 1.66	0.698
Fatty degeneration (grade)			
Supraspinatus	1.41 ± 0.88	1.25 ± 0.91	0.316
Infraspinatus	0.47 ± 0.57	0.44 ± 0.66	0.814
Subscapularis	0.50 ± 0.66	0.58 ± 1.05	0.631

^a^Results of independent *t*-test or chi-square test between group comparison.

^b^Small tear (≤ 1 cm), medium (1–3 cm), large (3–5 cm), and massive (> 5 cm).

### Primary outcome

**Fig. 2:**
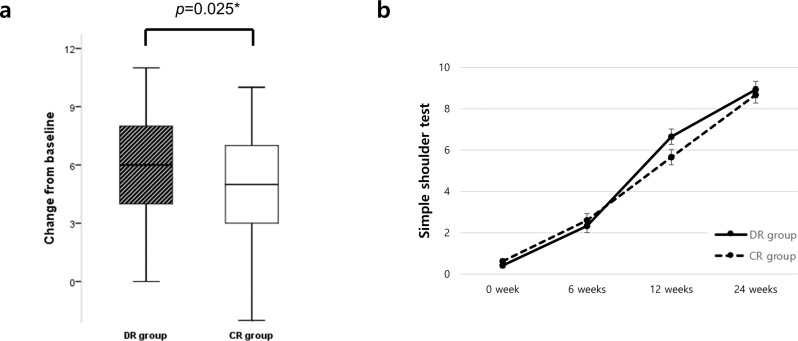
Primary outcome. **a** Changes of Simple shoulder test between baseline and 12 weeks postoperatively. *p* value for the primary outcome generated by the independent *t*-test. The box is shown as median (center line) and first and third quartiles, with bars representing minimum and maximum. **b** Simple shoulder test at various time points. The error bars represent the standard error.

There was a significant difference in primary outcome between the DR and CR groups (6.24 ± 2.63 vs. 5.04 ± 2.86, respectively; *p* = 0.025) (Table 2, Fig. 2a). Although the changes in SST score were greater in the DR group than in the CR group at all times, there was no significant group×time interaction (Table 2, Fig. 2b). Additionally, comparing the change in SST during 12 weeks between two groups according to the tear size category, the medium size tear group showed a significant difference (Supplementary Fig. a).

**Table 2:** Primary outcome analysis of SST.

	DR group (*n* = 55)	CR group (*n* = 53)	*p* value^a^	Time×group interaction^b^
SST				0.123
Baseline	0.42 ± 0.88	0.62 ± 0.86	0.224	
6 weeks	2.33 ± 2.20	2.60 ± 2.58	0.550	
12 weeks	6.65 ± 2.57	5.66 ± 2.84	0.059	
24 weeks	8.93 ± 2.68	8.68 ± 3.16	0.661	
Δbaseline-6weeks	1.76 ± 2.32	1.46 ± 2.20	0.541	
Δbaseline-12weeks	6.24 ± 2.63	5.04 ± 2.86	0.025	
Δbaseline-24weeks	8.66 ± 2.90	7.80 ± 3.14	0.226	

*SST* Simple Shoulder Test.

^a^Results of independent *t*-test between group comparison.

^b^Results of repeated measures ANOVA for group by time effect.

### Secondary outcome

**Fig. 3:**
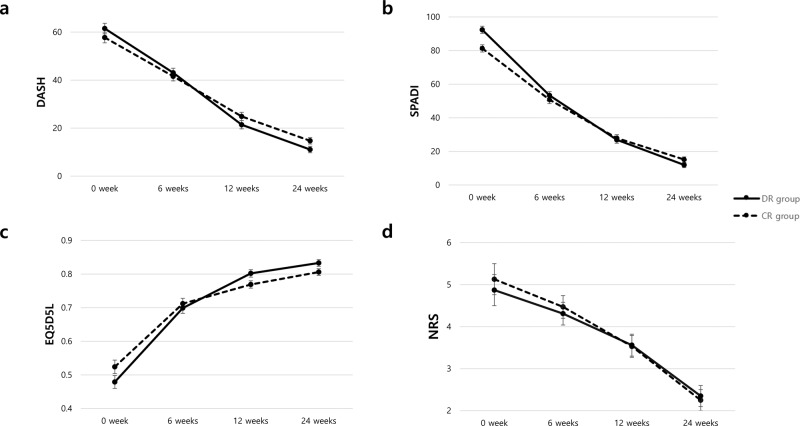
Self-reported outcomes. **a** DASH, **b** SPADI, **c** EQ5D5L, and **d** NRS. The error bars represent the standard error.

The secondary outcomes are presented in Table 3 and Table 4. There were no significant differences between the groups at baseline, except for the SPADI score (*p* = 0.001). Compared to the participants in the CR group, those in the DR group had greater improvements in the DASH (Δ24 weeks), SPADI (Δ6, 12, and 24 weeks), and EQ5D5L (Δ12 and 24 weeks) scores. Additionally, there were significant group × time interactions for the DASH, SPADI, and EQ5D5L scores (p = 0.040, =0.001, and =0.016, respectively) (Table 3, Fig. 3). There was no significant difference between the groups and group × time interaction in terms of pain relief (Table 3, Fig. 3). As a result of subgroup analysis to determine the difference between the two groups according to tear size, DASH, SPAID, and EQ5D5L showed significant differences in medium-sized tear group (Supplementary Fig. b–d). No group differences or group×time interactions were observed for the objective functional outcomes, including ROM, muscle strength, and handgrip strength (Table 4). Both groups had significant improvement in all outcomes over time (all *p* < 0.001, data not shown).

**Table 3:** Secondary outcome analysis of DASH, SPADI, EQ5D5L, and NRS.

	DR group (*n* = 55)	CR group (*n* = 53)	*p* value^a^	Time×group interaction^b^
DASH				0.040
Baseline	61.50 ± 16.36	57.77 ± 16.03	0.234	
6 weeks	43.08 ± 14.53	41.58 ± 13.08	0.575	
12 weeks	21.42 ± 10.40	24.88 ± 14.44	0.158	
24 weeks	11.08 ± 7.45	14.75 ± 11.75	0.057	
Δbaseline-6weeks	−18.41 ± 17.46	−16.18 ± 17.29	0.506	
Δbaseline-12weeks	−40.08 ± 16.73	−32.89 ± 20.72	0.050	
Δbaseline-24weeks	−50.42 ± 15.87	−43.02 ± 19.28	0.031	
SPADI				0.001
Baseline	92.36 ± 10.63	81.31 ± 19.67	0.001	
6 weeks	53.31 ± 18.28	50.76 ± 15.29	0.434	
12 weeks	26.90 ± 14.11	27.89 ± 15.98	0.734	
24 weeks	11.94 ± 10.08	15.18 ± 12.66	0.145	
Δbaseline-6weeks	−39.05 ± 21.93	-30.55 ± 18.78	0.033	
Δbaseline-12weeks	−65.46 ± 17.12	−53.42 ± 22.87	0.003	
Δbaseline-24weeks	−80.42 ± 13.19	−66.13 ± 22.50	<0.001	
EQ5D5L				0.016
Baseline	0.479 ± 0.131	0.524 ± 0.157	0.105	
6 weeks	0.699 ± 0.107	0.712 ± 0.126	0.567	
12 weeks	0.802 ± 0.053	0.769 ± 0.103	0.039	
24 weeks	0.833 ± 0.053	0.806 ± 0.091	0.067	
Δbaseline-6weeks	0.221 ± 0.151	0.188 ± 0.171	0.297	
Δbaseline-12weeks	0.324 ± 0.141	0.245 ± 0.177	0.012	
Δbaseline-24weeks	0.354 ± 0.135	0.282 ± 0.166	0.015	
NRS				0.878
Baseline	4.87 ± 2.54	5.13 ± 2.88	0.602	
6 weeks	4.31 ± 1.97	4.47 ± 1.98	0.670	
12 weeks	3.56 ± 1.98	3.53 ± 1.83	0.923	
24 weeks	2.35 ± 1.88	2.25 ± 1.80	0.778	
Δbaseline-6weeks	−0.56 ± 3.32	−0.66 ± 3.33	0.880	
Δbaseline-12weeks	−1.31 ± 3.01	−1.60 ± 3.25	0.626	
Δbaseline-24weeks	−2.53 ± 3.04	−2.89 ± 3.08	0.543	

*DASH* Disabilities of Arm, Shoulder and Hand Questionnaire, *SPADI* Shoulder Pain And Disability Index, *EQ5D5L* EuroQoL 5-Demension 5-Level questionnaire, *NRS* Numeric Rating Scale.

^a^Results of independent *t*-test between group comparison.

^b^Results of repeated measures ANOVA for group by time effect.

**Table 4:** Secondary outcome analysis of grip strength, ROM, and MMT.

	DR group (*n* = 55)				CR group (*n* = 53)				*p* value^a^
	Baseline	6 weeks	12 weeks	24 weeks	Baseline	6 weeks	12 weeks	24 weeks	Interaction
Grip strength	17.3 ± 8.7	20.5 ± 8.5	22.3 ± 8.4	25.7 ± 6.6	18.0 ± 8.4	21.8 ± 7.6	24.9 ± 7.5	25.7 ± 9.2	0.548
ROM (F)	-	96.8 ± 32.9	142.9 ± 27.8	166.2 ± 21.6	-	93.2 ± 33.9	132.4 ± 39.6	155.6 ± 38.0	0.466
ROM (AB)	-	88.9 ± 34.2	146.8 ± 35.7	169.3 ± 22.9	-	85.9 ± 36.5	132.2 ± 46.9	159.6 ± 41.4	0.570
ROM (IR)	-	37.9 ± 17.6	46.8 ± 18.0	53.2 ± 14.6	-	37.2 ± 19.1	44.3 ± 19.7	50.0 ± 16.3	0.955
ROM (ER)	-	37.6 ± 20.0	65.8 ± 17.9	80.9 ± 11.9	-	40.7 ± 19.0	59.8 ± 20.5	74.7 ± 16.6	0.074
MMT (AB)	-	40.3 ± 21.4	52.9 ± 23.5	90.5 ± 43.2	-	32.2 ± 24.0	50.3 ± 27.4	77.8 ± 42.6	0.564
MMT (ER)	-	49.1 ± 23.4	68.9 ± 25.1	88.5 ± 35.9	-	40.2 ± 15.8	59.8 ± 21.7	77.1 ± 29.4	0.885

*ROM* Rang of Motion, *MMT* Manual Muscle Test, *F* flexion, *AB* abduction, *IR* internal rotation, *ER* external rotation.

^a^Results of repeated measures ANOVA for group by time effect.

### Participant satisfaction

**Fig. 4:**
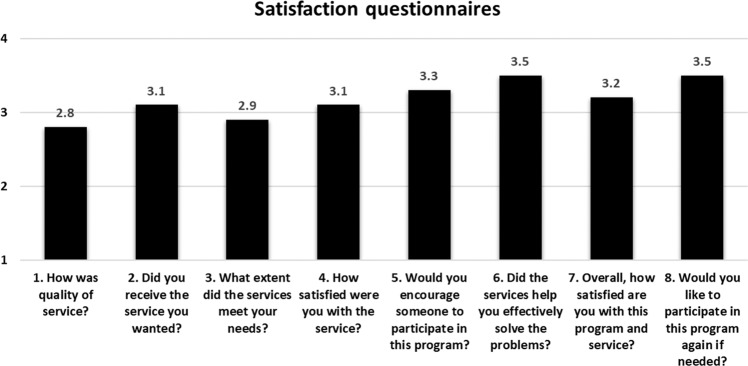
Results of the telerehabilitation satisfaction questionnaire. The average satisfaction levels are indicated above the bars.

Participants in the DR group answered eight questions related to their satisfaction level on a scale of 1 (Not at all) to 4 (Extremely). The 36 respondents reported a high level of satisfaction with the use of the digital healthcare system and services, with an average score of 3.2 out of 4. The highest-scoring items were “Did the services help you to effectively solve the problems?” and “Would you like to participate in this program again, if needed?” (Fig. 4).

## Discussion

We compared the effectiveness of AR- and brochure-based home exercise in patients after RCR. Although there was significantly greater change in the SST score in the DR group compared to the CR group, the group×time interaction was not significant. However, compared to the CR group, the DR group exhibited greater improvements in the DASH, SPADI, and EQ5D5L scores over time. In subgroup analysis, these significant differences were found in the medium-sized tear group, suggesting that AR-based rehabilitation is effective in this group. However, there were no differences between the groups in terms of postoperative pain, ROM, muscle strength, and handgrip strength.

Our results indicate significant improvement in the self-reported functional outcome with AR-based rehabilitation compared to conventional rehabilitation. Notably, there were significant differences between the groups in terms of disability (i.e., SPADI score at 12 weeks [*p* = 0.004] and 24 weeks [*p* < 0.001]), self-care (i.e., EQ5D5L at 12 weeks [*p* = 0.005] and 24 weeks [*p* = 0.010]), and usual activities (i.e., EQ5D5L at 12 weeks [*p* = 0.003] and 24 weeks [*p* = 0.001]) (Supplementary Table). Additionally, there were significant group×time interactions for disability (SPADI), self-care (EQ5D5L), and usual activities (EQ5D5L) (*p* = 0.001, =0.011, and =0.001, respectively). However, no differences were observed between groups in their pain-related subscales scores. Thus, an AR-based digital healthcare system is effective for improvement in shoulder function, such as the return to activities of daily living and improvement of the quality of life, after RCR.

The DASH, SPADI, and EQ5D5L are patient-reported outcome measurements (PROMs) that evaluate patient’s perception of their own health. Based on the abovementioned results, participants in the DR group perceived themselves to be well-recovered, which was related to satisfaction with rehabilitation services and self-efficacy. The DR group participants reported high satisfaction levels with the use of the digital healthcare system in terms of real-time feedback, programs supported by experts, and rehabilitation service without transportation concerns. These findings are in line with those of a previous study that showed a high satisfaction level with a home-based strengthening and stabilization system after shoulder surgery^15^. PROMs are a major target for digital healthcare systems to achieve better outcomes.

Recently, there is increasing demand for telerehabilitation (i.e., delivery of rehabilitation services directly to patients’ homes using online platforms, particularly because of the COVID-19 pandemic). The use of telerehabilitation can reduce healthcare-related costs and improve the accessibility of rehabilitation services in rural and remote areas^16^. Technologies used in telerehabilitation are diverse, such as videoconference^17–19^, mobile applications^20,21^, and virtual reality (VR)^22,23^. Of these, AR, derived from VR, provides a better sense of reality by blending the real environment and virtual objects. AR-based rehabilitation provides better proprioceptive feedback through interactions with the surrounding environment^24^. Previous studies have reported that AR-based rehabilitation was effective for maintaining balance and preventing falls in the geriatric population^25,26^ and lower and upper extremity function in stroke patients^14,27^. Similarly, the current trial showed that AR-based rehabilitation was effective for the improvement of shoulder function after RCR.

Digital healthcare for postoperative rehabilitation including RCR, is still emerging area and previously published studies have mainly demonstrated the feasibility after lower extremity surgery, with some studies showing superior results compared to conventional rehabilitation. are demonstrating feasibility^28–30^. On the other hand, studies evaluating the use of digital healthcare rehabilitation system after RCR were relatively rare. Comparing the effect of this study with previously reported MCID (MCID for SST, 2.33^31^; DASH, 10.2^32^; SPADI 15.4^32^; EQ5D5L, range from 0.03 to 0.54 depending on the estimation procedures^33^), the mean changes for SST, DASH, SPAID and EQ5D5L in this study during 12 weeks postoperatively were clinically significant in both groups (Table 2 and Table 3). A recently published study by Correia et al. found that the digital therapeutic group was not superior to the conventional therapy group in terms of treatment, which is different from our study results^34^. The discrepancies in the study results might be due to differences in the participants, rehabilitation protocols, and digital devices. The previous study included participants aged ≥18 years, and excluded those with a complex cuff tear (involving more than one tendon or a massive tear). That is, the study was conducted among participants who had a relatively high functional level at baseline compared to our study sample.

The number of dropouts differed between the groups. Most dropouts occurred in the DR group at the time of 6 weeks postoperatively. Seven participants discontinued the intervention due to refusal of device installation. The main reasons were reluctance to have someone visit their home, particularly during the COVID-19 pandemic, and install a motion tracking device in their home. These reasons for withdrawal were observed in the DR group only, suggesting a need to improve the digital healthcare system in the future. Two participants in the CR group discontinued the intervention because of falls and medical reasons; the reasons were not related to our intervention. Falls occurred during routine activities, not during exercise, and the medical reasons for discontinuation (e.g., vertigo) were related to the underlying disease. There were no significant adverse events in the study, suggesting that AR-based rehabilitation is clinically safe.

This study had several limitations. First, the outcomes were measured postoperatively. Because the baseline assessment was performed between 1 and 14 days after surgery, there was a lack of data on the objective physical outcomes at baseline, which might have affected out study results. Second, there was difference between two groups in SPADI score at baseline despite randomly allocation. Nevertheless, the DR group showed greater improvement at each time point than the CR group, and the group×time interaction showed significant results. Third, although participants in both groups were provided instructions to perform the exercises for the same duration, the actual exercise duration differed between the group, which could have affected out study results. These differences are due to differences in the methods of counting the number of exercise days. The exercise quantity was recorded by the Internet server after the entire session was completed in the DR group, participants in the CR group recorded whether the exercises were performed and the number of repetitions in an exercise diary.

In conclusion, AR-based digital healthcare rehabilitation showed better improvement than conventional rehabilitation in terms of shoulder function and quality of life after RCR. No adverse events were noted. Participants in the DR group reported high satisfaction rates. Our results suggest that AR-based rehabilitation is effective and safe compared to conventional rehabilitation.

## Methods

### Study design

This prospective, single-center, assessor-blinded, randomized controlled trial, randomly assigned participants to digital healthcare rehabilitation group (DR group) and conventional rehabilitation group (CR group) (Fig. 1). Both groups received the intervention for 12 weeks and were followed for up to 24 weeks. Participants in the DR group performed brochure-based exercises for 6 weeks followed by AR-based exercises for 6 weeks, whereas participants in the CR group performed brochure-based exercises for 12 weeks.

**Fig. 5:**
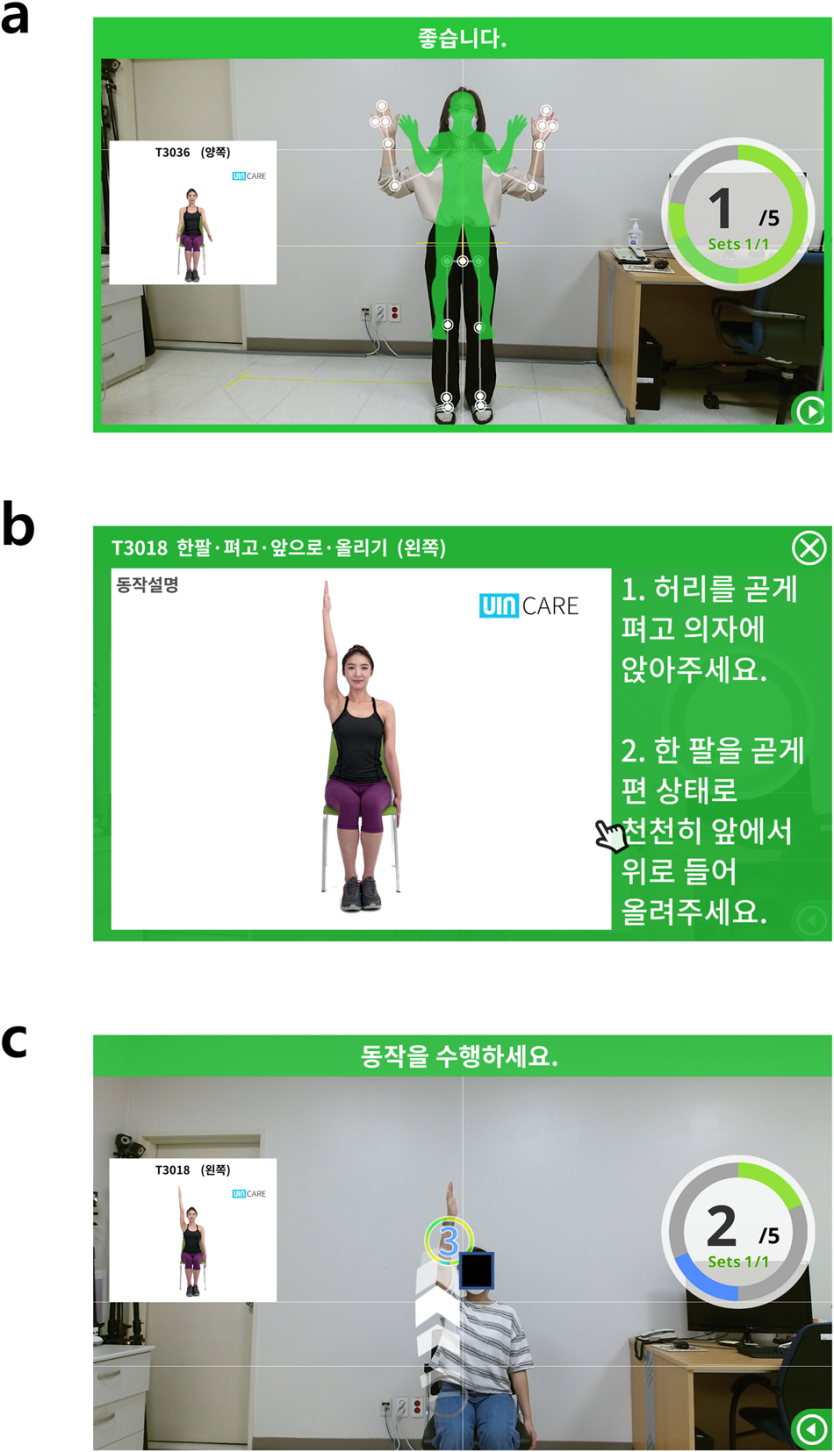
The digital healthcare system. **a** Before starting an exercise, the participants’ body was recognized on the monitor. **b** Avatars and written summaries of the motions were displayed on the monitor. **c** Participants followed the motions displayed on the monitor, which were captured by a camera. Consent was obtained from the participant to use these images.

The study protocol was approved by the Institutional Review Board of Seoul National University at Bundang Hospital (SNUBH) (IRB no.: B-2005-612-001), and it was registered on ClinicalTrials.gov (approval ID: NCT04511377, http://clinicaltrials.gov/ct2/show/NCT04511377) on August 10, 2020 under the title “Rehabilitation Exercise Using Digital Healthcare System in Patients with Rotator Cuff Repair”. Written informed consent was obtained from the participants. With respect to Fig. 5, the authors affirm that participants provided informed consent for publication. The study was conducted in accordance with the Declaration of Helsinki. The detailed protocol has been published previously^35^.

### Participants

We recruited participants who underwent RCR at the orthopedic department of SNUBH (Seongnam-si, Korea) between August 2020 to November 2021. The medical records of patients were searched 1-3 days postoperatively to determine whether they fulfilled the eligibility criteria^35^. The study included participants aged ≥ 50 years who underwent RCT alone or with biceps tenotomy, acromioplasty, or labral repair, and who were able to be discharged home. We excluded participants with a history of prior surgery on the affected shoulder, severe neurological deficits, an infection in the affected shoulder, a history of reverse total shoulder arthroplasty, or total shoulder arthroplasty, or the inability to exercise due to severe comorbidities.

### Randomization and blinding

Participants were randomly allocated at a 1:1 ratio to the DR or CR group using SAS version 9.4 program (SAS Institute, Cary, NC, USA). Randomization was performed using a computer-generated sequence with a block size of four. An unblinded coordinator, who did not participate in enrollment and assessment, allocated the participants. The nature of the study did not allow blinding of participants. The assessments were performed by two experienced investigators who were blinded to the groups and the participants were instructed not to reveal their group allocation during assessments.

### Interventions

Participants were enrolled in a 12-week rehabilitation program, consisting of three phases: on-brace phase (from immediate to 6 weeks postoperatively), off-brace phase (6-9 weeks postoperatively), and active mobilization phase (9–12 weeks postoperatively)^36,37^. Prior to discharge, the participants were educated by an experienced physical therapist and provided with a brochure that illustrated the exercises and included brief written summaries of each motion. The exercise protocol has been published previously^35^. Both groups performed the same type and duration of exercises. In addition, usual care, such as medication or physical therapy for pain relief, were allowed in both groups.

Participants in the DR group began with brochure-based exercises in the on-brace phase. During this phase, low-intensity whole body exercise and upper extremity mobilization exercise were performed, similar to the CR group. Next, in the off-brace phase, an AR-based digital healthcare system (UINCARE Home+ UINCARE Corp., Seoul, Korea) was installed at the participants’ homes by a technician on the research team. Technical support was also provided if a monitor or Wi-Fi was unavailable. Installation took about 10–20 min, after installation, the technician showed participants how to use the system for 20–30 min and, in turns, participants followed that. The digital healthcare system is designed with an easy and simple interface that allows participants to activate the program, click the “Today’s Session button”, and start the exercises for the day. Participants received a telephone number of the manufacturer in case of technical error or unstable connections.

The digital healthcare system consists of four components: software for the rehabilitation program, a three-dimensional-depth camera (Xbox One Kinect for Windows^®^, Microsoft, Redmond, WA, USA), a computer, and a display (TV or monitor) device. A three-dimensional camera sensor, a universal serial bus plug-and-play device that translates the scene geometry into depth information, tracks the movements of 25 joints of the upper and lower extremities. Before starting an exercise, participants were positioned to recognize their bodies on the screen. Each exercise was displayed on the screen as an avatar’s actual motion and written summaries. When participants performed the exercise, they were provided with real-time feedback on the screen (Fig. 5). After each session was completed, the accuracy and completion of the exercise were displayed on the screen to provide achievement to the participants. In addition, the exercise performance and movement accuracy were also recorded on an Internet server and reviewed by a physician. Participants received detailed feedback on their performance in the outpatient clinic around 6 and 12 weeks postoperatively.

Participants in the CR group performed brochure-based home exercises, as is the standard rehabilitation protocol for patients with RCR at our hospital^35^. From the on-brace phase to the active mobilization phase, participants were instructed to perform 3–5 sets of exercises with 10 repetitions of each set per day, similar to the DR group. Participants were asked to maintain an exercise diary and their condition was checked by weekly telephone call. If participants complain of pain during exercise, and the pain is tolerable, encouraging them to perform the exercise regularly.

### Outcomes

Outcomes were assessed at 0 (baseline), 6, 12, and 24 weeks postoperatively. Because the baseline assessment was performed during the on-brace phase, range of motion (ROM) and muscle strength were not measured.

The primary outcome was the change in simple shoulder test (SST) score between baseline and 12 weeks postoperatively. The SST is self-reported shoulder-specific questionnaire that measure functional limitation of shoulder, such as pain, ROM, and strength^31,38^. The SST consists of 12 questions, rated as “yes” (1) or “no” (0), and each question asking whether specific activity can be performed. The total score ranges from 0 (worst) to 12 (best)^39^. It shows high test-retest reliability (intraclass correlation coefficient > 0.90)^40,41^.

The secondary outcomes included the shoulder function with disabilities of arm, shoulder and hand (DASH) and shoulder pain and disability index (SPADI); quality of life with EuroQoL 5-Dimension 5-Level questionnaire (EQ5D5L); pain measured using a numeric pain rating scale (NRS); and objective functional outcomes (e.g., ROM, muscle strength, and handgrip strength).

The DASH survey consists of 30 questions related to difficulty in performing physical activities (21 items), severity of pain, activity-related pain, tingling sensation, weakness, and stiffness (5 items), and problems with social activities, work, sleep, and self-image (4 items). Each item is rated on a 5-point scale. The total scores ranges from 0 (no disability) to 100 (most severe disability)^42^. The SPADI consists of 13 questions related to pain (5 items) and disability (8 items). Each item is marked on a 10-point scale from 0 (no pain at all or no difficulty) to10 (worst pain imaginable or need for help). The total score ranges from 0 to 100^43^. The EQ5D5L comprises five dimensions: mobility, self-care, usual activities, pain/discomfort, and anxiety/depression. Each dimension has 5 levels: no, slight, moderate, severe, and extreme problems^44^. Pain intensity during activity on the affected side was measured using a NRS, rating from 0 (no pain) to 10 (worst pain imaginable)^45^. The active ROM on the affected side was measured within the pain-free range in four directions: shoulder forward flexion (0–180°), abduction (0–180°), external rotation (0–90°), and internal rotation (0–70°)^46^. Muscle strength of the affected shoulder during abduction and external rotation was assessed using a Lafayette Hand-held Dynamometer (Lafayette Instrument Co^®^, Lafayette, IN, USA)^47^. Handgrip strength on the affected side was measured using a Takei Handgrip Dynamometer (Takei Scientific Instruments Co., Ltd., Tokyo, Japan). Participants were asked to squeeze the dynamometer with maximal effort in a standing position with the elbow fully extended, without moving the hand and arm^48^.

The other recorded variables included acromion thickness, fatty degeneration of rotator cuff muscles (supraspinatus, infraspinatus, and subscapularis), and tear size based on preoperative magnetic resonance imaging (MRI) and perioperative findings. The acromion thickness was measured on the oblique sagittal view by MRI. Fatty degeneration was graded on a scale of 0 to 4 according to the Goutallier classification system^49^. The size of the rotator cuff tear was measured in two planes (anterior to posterior [AP] and medial to lateral [retraction]) on a sagittal T2-weighted MRI sequence. Perioperative tear size was measured intraoperatively and categorized similar to a previous study by DeOrio and Cofield^50^: small, ≤1 cm; medium, 1–3 cm; large, 3–5 cm; and massive, >5 cm or involving more than one tendon.

### Sample size

The minimal clinically important difference in SST score for patients with a rotator cuff tear was reported as 4.3^51^. The mean SST score 3 months postoperatively was 6.34 (standard deviation^52^ = 3.7)^53^. The minimum sample size was calculated based on a 2.15-point difference in SST score (50% of the minimal clinically important difference, MCID), SD of 3.7, probability of a type 1 error of 5%, and statistical power of 80%. A minimum of 49 participants were required in each group. Assuming 15% attrition per group; a total of 115 participants were required to be enrolled.

### Statistical analysis

Statistical analyses were performed using an intention-to-treat approach. Missing outcome data were addressed using multiple imputations^54^. Differences in the clinical and demographic variables of the two groups were assessed using an independent *t*-test or chi-square test. The primary and secondary outcomes were compared between the groups using an independent *t*-test for each time point and changes from baseline. Additionally, a repeated measures analysis of variance was used to analyze the differences in outcomes between the groups over time. *P* < 0.05 was considered statistically significant. Statistical analyses were performed using IBM SPSS software (version 23.0 for Windows; IBM Corp., Armonk, NY, USA).

### Reporting summary

Further information on research design is available in the Nature Research Reporting Summary linked to this article.

## Supplementary information

Reporting Summary

Supplementary file

## Data Availability

The data that support the reported findings are available from the corresponding authors upon reasonable request.
