# Supplementary material for: A randomized controlled trial of postoperative rehabilitation using digital healthcare system after rotator cuff repair
Source: NPJ Digit Med. 2023 May 23;6:95. doi: 10.1038/s41746-023-00842-7 (PMC10204020; doi:10.1038/s41746-023-00842-7)
Supplement: Supplementary file 1 — Reporting Summary [file 41746_2023_842_MOESM1_ESM.pdf]

nature portfolio

Corresponding author(s): Jae-young Lim, Ji Hye Hwang

Last updated by author(s): May 1, 2023

Reporting Summary

Nature Portfolio wishes to improve the reproducibility of the work that we publish. This form provides structure for consistency and transparency in reporting. For further information on Nature Portfolio policies, see our Editorial Policies and the Editorial Policy Checklist.

Statistics

For all statistical analyses, confirm that the following items are present in the figure legend, table legend, main text, or Methods section.

n/a	Confirmed	
☐	☒	The exact sample size (n) for each experimental group/condition, given as a discrete number and unit of measurement
☐	☒	A statement on whether measurements were taken from distinct samples or whether the same sample was measured repeatedly
☐	☒	The statistical test(s) used AND whether they are one- or two-sided<br>*Only common tests should be described solely by name; describe more complex techniques in the Methods section.*
☐	☒	A description of all covariates tested
☐	☒	A description of any assumptions or corrections, such as tests of normality and adjustment for multiple comparisons
☐	☒	A full description of the statistical parameters including central tendency (e.g. means) or other basic estimates (e.g. regression coefficient) AND variation (e.g. standard deviation) or associated estimates of uncertainty (e.g. confidence intervals)
☒	☐	For null hypothesis testing, the test statistic (e.g. F, t, r) with confidence intervals, effect sizes, degrees of freedom and P value noted<br>*Give P values as exact values whenever suitable.*
☒	☐	For Bayesian analysis, information on the choice of priors and Markov chain Monte Carlo settings
☒	☐	For hierarchical and complex designs, identification of the appropriate level for tests and full reporting of outcomes
☒	☐	Estimates of effect sizes (e.g. Cohen's d, Pearson's r), indicating how they were calculated

Our web collection on statistics for biologists contains articles on many of the points above.

Software and code

Policy information about availability of computer code

Data collection	Microsoft office Excel
Data analysis	SPSS version 23

For manuscripts utilizing custom algorithms or software that are central to the research but not yet described in published literature, software must be made available to editors and reviewers. We strongly encourage code deposition in a community repository (e.g. GitHub). See the Nature Portfolio guidelines for submitting code & software for further information.

Data

Policy information about availability of data

All manuscripts must include a data availability statement. This statement should provide the following information, where applicable:

- Accession codes, unique identifiers, or web links for publicly available datasets
- A description of any restrictions on data availability
- For clinical datasets or third party data, please ensure that the statement adheres to our policy

The datasets are not publicly available pending further research and/or analysis, but may be made available upon reasonable request to the corresponding author after ethical permission.

Human research participants

Policy information about studies involving human research participants and Sex and Gender in Research.

Reporting on sex and gender	This study was designed to recruit both sexes. Table 1 presented that both sexes were evenly distributed between two groups. In the previous studies, sex-based analysis is unnecessary because preoperative status (i.g. tear size, preoperative functional state) rather than sex affects the outcomes.
Population characteristics	The study presented all covariate in Table 1.
Recruitment	In this trial, participants were recruited by screening medical record charts of patients who underwent rotator cuff surgery in same hospital. There is a possibility that the place of residence of the participants acts as a bias: Residence may be related to income, lifestyle, and health concerns.
Ethics oversight	Institutional Review Board of Seoul National University at Bundang Hospital

Note that full information on the approval of the study protocol must also be provided in the manuscript.

Field-specific reporting

Please select the one below that is the best fit for your research. If you are not sure, read the appropriate sections before making your selection.

☐ Life sciences ☒ Behavioural & social sciences ☐ Ecological, evolutionary & environmental sciences

For a reference copy of the document with all sections, see nature.com/documents/nr-reporting-summary-flat.pdf

Behavioural & social sciences study design

All studies must disclose on these points even when the disclosure is negative.

Study description	The study aims to examine the effect of short-term rehabilitation exercise support using digital healthcare system (Uincare homeplus) in the patients with rotator cuff repair surgery. The study is a two-arm prospective randomized controlled study comparing the effect of rehabilitation exercise digital healthcare system at home with conventional brochure-based home exercise.
Research sample	Research sample was recruited among patients who underwent rotator cuff surgery in Seoul National University at Bundang Hospital.
Sampling strategy	Sample size was calculated based on previous studies. The minimal clinically important difference of the primary outcome, SST, was 4.3, and the mean SST score 3 months postoperatively was 6.34, with a standard deviation of 3.7. Therefore, assuming that the type 1 error is 5% and the statistical power is 80%, a minimum of 49 participants was required for each group, and a total of 115 participants were enrolled considering a dropout of 15%.
Data collection	Data collection was performed by an experienced investigators who did not participate in the group allocation at 0, 6, and 12 weeks postoperatively. Beside patient-reported outcome measurements, ROM was measured using goniometer, muscle strength was measured using Lafayetter Hand-held Dynamometer, handgrip strength was measured using Takei handgrip dynamometer.
Timing	Data collection started from August 2020 and stopped at November 2021.
Data exclusions	A total of 230 patients who underwent rotator cuff repair were screened for eligibility and half of them excluded by exclusion criteria. Among 115 participants, 7 participants who withdrew consent before intervention were excluded from ITT analysis, which is not included in the exclusion criteria.
Non-participation	As presented Figure 1, 5 participants were withdrew consent during intervention, 7 participants decline to install device, 1 participants withdrew for self-reception of a recovery, 2 participants were moved out, AND 1 participants withdrew for dissatisfied allocation, medical reason, and experience of fall, respectively.
Randomization	Participants were randomly allocated at a 1:1 ratio to the DR or CR group using SAS version 9.4 program (SAS Institute, Cary, NC, USA). Randomization was performed using a computer-generated sequence with a block size of four. An unblinded coordinator, who did not participate in enrollment and assessment, allocated the participants. The nature of the study did not allow blinding of participants. The assessments were performed by two experienced investigators who were blinded to the groups and the participants were instructed not to reveal their group allocation during assessments.

Reporting for specific materials, systems and methods

We require information from authors about some types of materials, experimental systems and methods used in many studies. Here, indicate whether each material, system or method listed is relevant to your study. If you are not sure if a list item applies to your research, read the appropriate section before selecting a response.

Materials & experimental systems

n/a	Involved in the study
☒	☐ Antibodies
☒	☐ Eukaryotic cell lines
☒	☐ Palaeontology and archaeology
☒	☐ Animals and other organisms
☐	☒ Clinical data
☒	☐ Dual use research of concern

Methods

n/a	Involved in the study
☒	☐ ChIP-seq
☒	☐ Flow cytometry
☒	☐ MRI-based neuroimaging

Clinical data

Policy information about clinical studies

All manuscripts should comply with the ICMJE guidelines for publication of clinical research and a completed CONSORT checklist must be included with all submissions.

Clinical trial registration	NCT04511377
Study protocol	The study aims to examine the effect of short-term rehabilitation exercise support using digital healthcare system (Uincare homeplus) in the patients with rotator cuff repair surgery. The study is a two-arm prospective randomized controlled study comparing the effect of rehabilitation exercise digital healthcare system at home with conventional brochure-based home exercise. Simple Shoulder Test (SST), Pain (using Numerical rating scale), shoulder range of motion (ROM), Disability of Arm, Shoulder and Hand (DASH), Shoulder Pain and Disability Index (SPADI), quality of life using EQ-5D will be evaluation on enrollment, 6-weeks, 12-weeks and 24-weeks after enrollment.
Data collection	Data collection was performed by an experienced investigators who did not participate in the group allocation at 0, 6, and 12 weeks postoperatively.
Outcomes	Simple Shoulder Test (SST), Pain (using Numerical rating scale), shoulder range of motion (ROM), Disability of Arm, Shoulder and Hand (DASH), Shoulder Pain and Disability Index (SPADI), quality of life using EQ-5D
