# Supplementary material for: A randomized controlled trial of postoperative rehabilitation using digital healthcare system after rotator cuff repair
Source: NPJ Digit Med. 2023 May 23;6:95. doi: 10.1038/s41746-023-00842-7 (PMC10204020; doi:10.1038/s41746-023-00842-7)
Supplement: Supplementary file 2 — Supplementary file [file 41746_2023_842_MOESM2_ESM.pdf]

Supplementary Table. Subscale analysis of SPADI and EQ5D5L.

	DR group (n=55)	CR group (n=53)	*p* value[a]	Time×group interaction[b]
SPADI (pain)				0.548
Baseline	28.78±18.57	25.42±16.97	0.328	
6 weeks	24.07±9.48	22.19±9.05	0.294	
12 weeks	16.04±7.40	15.00±7.73	0.478	
24 weeks	8.20±6.86	8.25±6.54	0.972	
Δbaseline-6weeks	-4.71±17.11	-3.23±14.18	0.626	
Δbaseline-12weeks	-12.75±18.74	-10.42±17.71	0.508	
Δbaseline-24weeks	-20.58±18.44	-17.17±17.20	0.323	
SPADI (disability)				0.001
Baseline	76.89±8.34	67.62±17.15	0.001	
6 weeks	43.29±17.15	40.59±15.29	0.389	
12 weeks	18.84±12.84	20.28±14.55	0.586	
24 weeks	7.33±7.68	10.96±10.86	0.048	
Δbaseline-6weeks	-33.6±18.94	-27.04±18.48	0.071	
Δbaseline-12weeks	-58.06±15.24	-47.34±21.87	0.004	
Δbaseline-24weeks	-69.56±11.02	-56.66±20.95	<0.001	
EQ5D5L (mobility)				0.338
Baseline	1.27±0.56	1.36±0.56	0.427	
6 weeks	1.27±0.56	1.15±0.41	0.199	
12 weeks	1.11±0.31	1.12±0.51	0.456	
24 weeks	1.16±0.37	1.21±0.41	0.561	
Δbaseline-6weeks	0.00±0.75	-0.21±0.74	0.150	
Δbaseline-12weeks	-0.16±0.63	-0.19±0.78	0.855	
Δbaseline-24weeks	-0.11±0.60	-0.15±0.72	0.742	
EQ5D5L (self-care)				0.011
Baseline	4.46±0.98	4.04±1.14	0.044	
6 weeks	2.78±1.01	2.70±1.01	0.668	
12 weeks	1.58±0.60	1.85±0.69	0.034	

24 weeks	1.27±0.49	1.53±0.67	0.026	
Δbaseline-6weeks	-1.67±1.36	-1.34±1.30	0.197	
Δbaseline-12weeks	-2.87±1.09	-2.19±1.40	0.005	
Δbaseline-24weeks	-3.18±1.16	-2.51±1.46	0.010	
EQ5D5L (usual activities)				0.001
Baseline	4.53±0.98	3.93±1.25	0.007	
6 weeks	2.67±0.92	2.53±1.01	0.440	
12 weeks	1.73±0.73	1.85±0.63	0.358	
24 weeks	1.44±0.71	1.68±0.83	0.105	
Δbaseline-6weeks	-1.86±1.16	-1.40±1.35	0.061	
Δbaseline-12weeks	-2.80±1.15	-2.08±1.31	0.003	
Δbaseline-24weeks	-3.09±1.14	-2.25±1.36	0.001	
EQ5D5L (pain/discomport)				0.473
Baseline	2.98±0.93	2.85±0.91	0.455	
6 weeks	2.53±0.63	2.42±0.72	0.391	
12 weeks	2.22±0.42	2.32±0.61	0.310	
24 weeks	1.86±0.56	1.81±0.56	0.688	
Δbaseline-6weeks	-0.46±1.07	-0.43±0.99	0.918	
Δbaseline-12weeks	-0.76±0.98	-0.53±1.01	0.222	
Δbaseline-24weeks	-1.13±0.94	-1.04±0.94	0.622	
EQ5D5L (anxiety/depression)				0.129
Baseline	1.56±0.79	1.77±1.12	0.261	
6 weeks	1.66±0.84	1.53±0.77	0.420	
12 weeks	1.55±0.66	1.74±0.88	0.206	
24 weeks	1.27±0.49	1.57±0.87	0.034	
Δbaseline-6weeks	0.91±0.93	-0.25±1.24	0.113	
Δbaseline-12weeks	-0.02±0.95	-0.04±1.19	0.925	
Δbaseline-24weeks	-0.29±0.81	-0.21±1.06	0.647	

SPADI Shoulder Pain And Disability Index, *EQ5D5L* EuroQoL 5-Demension 5-Level

questionnaire.

[a] Results of independent t-test between group comparison.

[b] Results of repeated measures ANOVA for group by time effect.

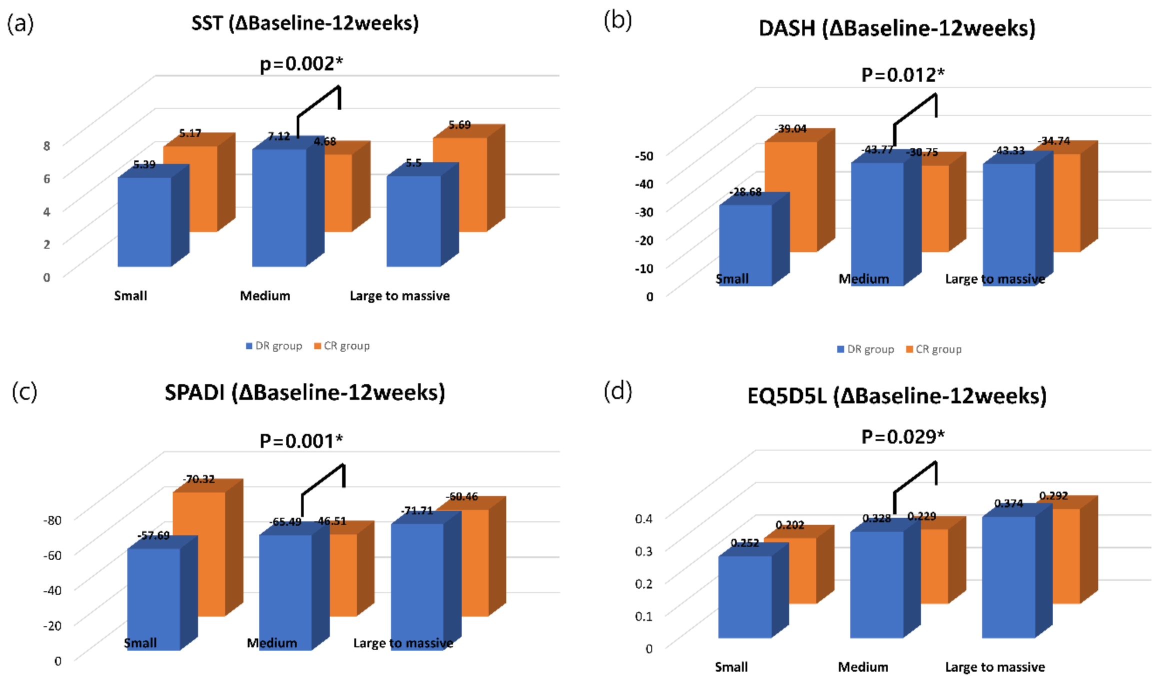

Supplementary Figure. Changes between baseline and 12 weeks postoperatively. (a) SST, (b) DASH, (c) SPADI, and (d) EQ5D5L according to the tear size. p value generated by the independent t-test.
